# Intestinal calcium and bile salts facilitate germination of *Clostridium difficile* spores

**DOI:** 10.1371/journal.ppat.1006443

**Published:** 2017-07-13

**Authors:** Travis J. Kochan, Madeline J. Somers, Alyssa M. Kaiser, Michelle S. Shoshiev, Ada K. Hagan, Jessica L. Hastie, Nicole P. Giordano, Ashley D. Smith, Alyxandria M. Schubert, Paul E. Carlson, Philip C. Hanna

**Affiliations:** 1 University of Michigan Medical School, Department of Microbiology and Immunology. Ann Arbor, Michigan, United States of America; 2 Center for Biologics Evaluation and Research, US Food and Drug Administration. Silver Spring, Maryland, United States of America; University of Texas Medical School at Houston, UNITED STATES

## Abstract

*Clostridium difficile* (*C*. *difficile*) is an anaerobic gram-positive pathogen that is the leading cause of nosocomial bacterial infection globally. *C*. *difficile* infection (CDI) typically occurs after ingestion of infectious spores by a patient that has been treated with broad-spectrum antibiotics. While CDI is a toxin-mediated disease, transmission and pathogenesis are dependent on the ability to produce viable spores. These spores must become metabolically active (germinate) in order to cause disease. *C*. *difficile* spore germination occurs when spores encounter bile salts and other co-germinants within the small intestine, however, the germination signaling cascade is unclear. Here we describe a signaling role for Ca^2+^ during *C*. *difficile* spore germination and provide direct evidence that intestinal Ca^2+^ coordinates with bile salts to stimulate germination. Endogenous Ca^2+^ (released from within the spore) and a putative AAA+ ATPase, encoded by *Cd630_32980*, are both essential for taurocholate-glycine induced germination in the absence of exogenous Ca^2+^. However, environmental Ca^2+^ replaces glycine as a co-germinant and circumvents the need for endogenous Ca^2+^ fluxes. *Cd630_32980* is dispensable for colonization in a murine model of *C*. *difficile* infection and *ex vivo* germination in mouse ileal contents. Calcium-depletion of the ileal contents prevented mutant spore germination and reduced WT spore germination by 90%, indicating that Ca^2+^ present within the gastrointestinal tract plays a critical role in *C*. *difficile* germination, colonization, and pathogenesis. These data provide a biological mechanism that may explain why individuals with inefficient intestinal calcium absorption (*e*.*g*., vitamin D deficiency, proton pump inhibitor use) are more prone to CDI and suggest that modulating free intestinal calcium is a potential strategy to curb the incidence of CDI.

## Author summary

The anaerobic, spore-forming bacterium *Clostridium difficile* (*C*. *difficile*) is a prominent pathogen in hospitals worldwide and the leading cause of nosocomial diarrhea. Numerous risk factors are associated with *C*. *difficile* infections (CDIs) including: antibiotics, advanced age, vitamin D deficiency, and proton pump inhibitors. Antibiotic use disrupts the intestinal microbiota allowing for *C*. *difficile* to colonize, however, why these other risk factors increase CDI incidence is unclear. Notably, deficient intestinal calcium absorption (*i*.*e*., increased calcium levels) is associated with these risk factors. In this work, we investigate the role of calcium in *C*. *difficile* spore germination. *C*. *difficile* spores are the infectious particles and they must become metabolically active (germinate) to cause disease. Here, we show that calcium is required for *C*. *difficile* germination, specifically activating the key step of cortex hydrolysis, and that this calcium can be derived from either within the spore or the environment. We also demonstrate that intestinal calcium is required for efficient spore germination *in vivo*, suggesting that intestinal concentrations of other co-germinants are insufficient to induce *C*. *difficile* germination. Collectively, these data provide a mechanism that explains the strong clinical correlations between increased intestinal calcium levels and risk of CDI.

## Introduction

The anaerobic spore-forming pathogen *Clostridium difficile* (*C*. *difficile*) is the leading cause of infectious nosocomial diarrhea, with 500,000 infections and 29,000 deaths in the U.S. annually [1]. *C*. *difficile* infection (CDI) typically occurs after antibiotic therapy disrupts the indigenous gut microbiota, allowing *C*. *difficile* colonization. Symptoms of CDI include diarrhea, pseudomembranous colitis, and toxic megacolon. Two *C*. *difficile* toxins, toxin A (TcdA) and toxin B (Tcd) are the primary cause of these pathologies causing epithelial cell death and inflammation [2]. While CDI symptoms are toxin-mediated, transmission and initiation of disease depend on the production of viable, metabolically dormant spores. *C*. *difficile* spores have a dehydrated core that contains cytoplasmic macromolecules (e.g. DNA, ribosomes) and 0.8-1M calcium-dipicolinic acid (Ca-DPA), which is biosynthesized during sporulation and required for the heat resistance of bacterial spores [3,4]. The spore core is surrounded by an inner membrane, a thick cortex of modified peptidoglycan, an outer membrane, a proteinaceous coat, and an outermost exosporium layer of proteins, lipids, and carbohydrates [5]. Collectively, these layers protect spores from harsh environmental conditions such as acidic pH, extreme temperature, and desiccation.

Bacterial spores become metabolically active, *i*.*e*., germinate, upon sensing specific small molecules, called germinants, in the environment. In the related *Bacillus spp*., spores contain numerous well-characterized germinant receptors on the inner membrane. These receptors interact with combinations of germinants including nucleotides and amino acids to initiate germination [6]. This process has been extensively studied in *Bacillus*, however, all sequenced *C*. *difficile* genomes lack the germinant receptors found in other spore-forming bacteria suggesting that the mechanism of germination in *C*. *difficile* is unique [7]. It is known that *C*. *difficile* germinates in response to co-germinants that include a combination of amino acids and bile salts; glycine and taurocholate (Tc) are the most efficient germinant combination [8,9]. It has been shown that Tc binds to CspC during germination [10,11], however, the receptors involved in the recognition of glycine or other amino acids have not been identified [12].

In *Bacillus spp*., germinant-receptor interactions induce slight hydration of the core causing a rapid release of monovalent cations (*e*.*g*., Na^+^, K^+^, H^+^) [13], followed by the release of Ca-DPA [14], and subsequent activation of cortex lytic enzymes (CLEs). CLEs degrade the cortex, initiating full core hydration and outgrowth of the vegetative bacteria. In *C*. *difficile*, SleC is the sole CLE that is essential for germination [15]. It is expressed as a zymogen that is activated by the subtilisin-like protease, CspB [16,17]. It is not known how CspC binding to Tc leads to the activation of CspB. CspC may directly interact with CspB, however, Tc-CspC interactions might also facilitate access of co-germinants to their receptors to initiate signaling to activate CspB. The mechanism by which co-germinants permeate the spore coat is not currently known, since the *C*. *difficile* genome does not contain homologues to the GerP proteins that perform this function in *Bacillus anthracis* [18]. Therefore, the signaling cascade leading to CspB activation is an important gap in the current knowledge of *C*. *difficile* germination. In this work, we provide direct evidence that calcium ions are a germination signal to activate CspB and can be derived from either endogenous or exogenous sources. We demonstrate that intestinal calcium is a key molecule for efficient germination in a murine model. This study provides novel insight into the Ca^2+^ signaling pathways controlling *C*. *difficile* germination and a biological mechanism that may help explain why inefficient intestinal Ca^2+^ absorption increases susceptibility to CDI.

## Results

### Exogenous calcium circumvents the glycine requirement for efficient *C*. *difficile* germination

**Fig 1:**
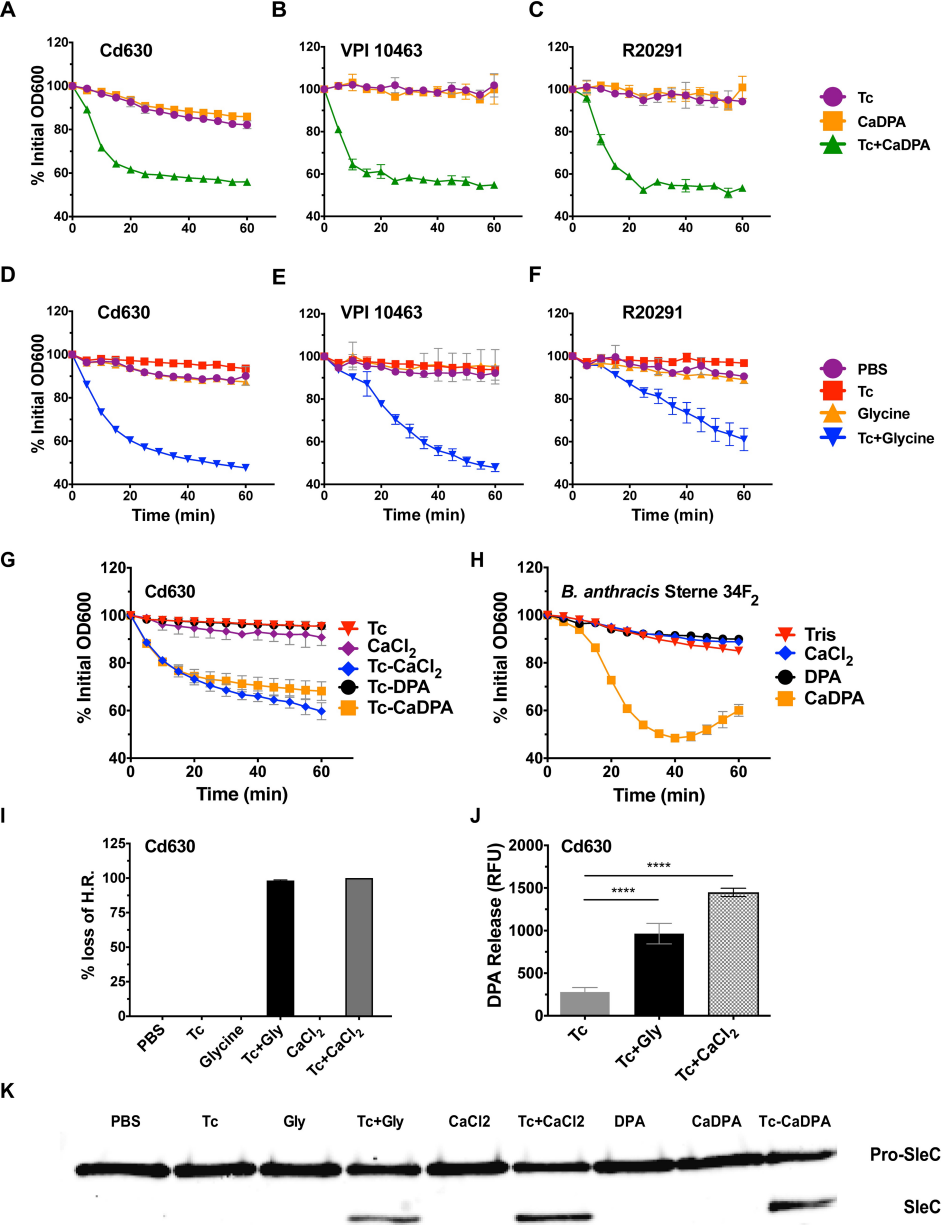
Exogenous calcium induces *C*. *difficile* germination in concert with taurocholate. Cd630, VPI 10463, or R20291 spores were incubated with the indicated combinations of 0.2% Tc, 60 mM Ca-DPA, 50 mM glycine, 60 mM CaCl_2_, or 60 mM DPA (A-G, I, J). Activation of SleC was assessed by western blot analyses. Cd630 spores were incubated for 15 minutes at 37°C with the indicated combinations of 1% Tc, 50 mM glycine, 60 mM CaCl_2_, 60 mM DPA, or 60 mM Ca-DPA. Spores were subsequently lysed and assayed for levels of pro-SleC and SleC (K). *Bacillus anthracis* strain Sterne 34F_2_ spores were incubated with the indicated combinations of either 60 mM CaCl_2_, 60 mM DPA, or 60 mM CaDPA (H). Germination was measured either by tracking the loss of OD_600_ over time (A-H), measuring loss of heat resistance at 37°C after 1 hour (I), or measuring release of DPA at 37°C after 1 hour (J). Germination assays were performed in triplicate. Germination assays and western blots are representative of three independent spore preps. Error bars are mean plus or minus SD. Statistical analysis was performed using one-way ANOVA. (****) p<0.0001.

The germination signal leading to CspB activation and cortex hydrolysis by SleC is currently unknown. In *Bacillus spp*., Ca-DPA released from the core functions as the germination signal to activate cortex hydrolysis. The addition of exogenous Ca-DPA induces spore germination by direct activation of the CLE CwlJ [19], circumventing the need for germinants or germinant receptors. To determine if exogenous Ca-DPA can induce *C*. *difficile* spore germination, spores of three toxigenic *C*. *difficile* strains were incubated with 60mM Ca-DPA. While none germinated in Ca-DPA alone [20], all three strains germinated efficiently (measured by loss of OD_600_) in Ca-DPA supplemented with 0.2% Tc (Fig 1A–1C). All three strains also germinated as expected in response to our positive control, Tc-Gly (Fig 1D–1F). However, two recent studies have demonstrated that cortex hydrolysis occurs prior to [21] or in the absence of DPA release [22], indicating that DPA is not essential for CLE activation. This led to our hypothesis that calcium ions serve as a germination signal in *C*. *difficile*. To test this hypothesis, Cd630 spores were incubated with Tc and either CaCl_2_, DPA, or Ca-DPA. As before, Tc-CaDPA induced full germination of *C*. *difficile* spores, however, Tc-CaCl_2_ also induced full germination of *C*. *difficile* spores in the absence of exogenous DPA (Fig 1G). Additionally, Tc-CaCl_2_-treated spores lost heat resistance properties of dormant spores and released internal stores of DPA (Fig 1I and 1J). In contrast, *B*. *anthracis* spores germinated as expected in response to Ca-DPA but were unable to germinate in response to CaCl_2_ alone (Fig 1H). We hypothesized that exogenous Ca^2+^ (or Ca-DPA) initiates cortex hydrolysis through SleC activation. To test this hypothesis, SleC activation was determined by western blot following incubation of Cd630 spores in PBS plus Tc, glycine, Tc-Gly, CaCl_2_, Tc-CaCl_2_, DPA, Ca-DPA, or Tc-CaDPA. While pro-SleC was present in all samples, activated SleC was only detected in samples incubated with Tc-Gly, Tc-CaCl_2_, or Tc-CaDPA (Fig 1K). These results indicate that SleC activation requires both Tc and an additional signal (*i*.*e*. glycine, calcium, or exogenous Ca-DPA) and that calcium induces germination through SleC activation. Since exogenous Tc-CaCl_2_ induced germination in *C*. *difficile*, and spores have large stores of internal calcium [23], these data suggest that calcium functions as a co-germinant, and can come from either exogenous or endogenous sources. Because both PBS only (untreated) and Tc only treated spores have identical germination phenotypes (Fig 1D–1F, 1I and 1K), Tc alone treatment was used as a negative control for remaining experiments.

To determine if other cations could stimulate *C*. *difficile* germination pathways, Cd630 spores were incubated with Tc and either CaCl_2_, Ca(NO^3^)_2_, Ca(C_2_H_3_O_2_)_2_, MgCl_2_, NaCl, ZnCl_2_, KCl, or LiCl. In addition to Tc-CaCl_2_, Tc-Ca(NO^3^)_2_, and Tc-Ca(C_2_H_3_O_2_)_2_ induced germination (~40% drop in OD_600_, which is considered ~100% germination), while Tc-MgCl_2_ induced minor germination (15% drop in OD_600_, S1 Fig). No other divalent cations were able to induce germination. *B*. *anthracis* spores were incubated with DPA and the cations listed above but were only able to germinate with Ca-DPA (S1 Fig). These data show that of the numerous cations tested only Ca^2+^ induced efficient germination in *C*. *difficile*.

### Calcium released from *C*. *difficile* spores is essential for germination in the absence of environmental Ca^2+^

**Fig 2:**
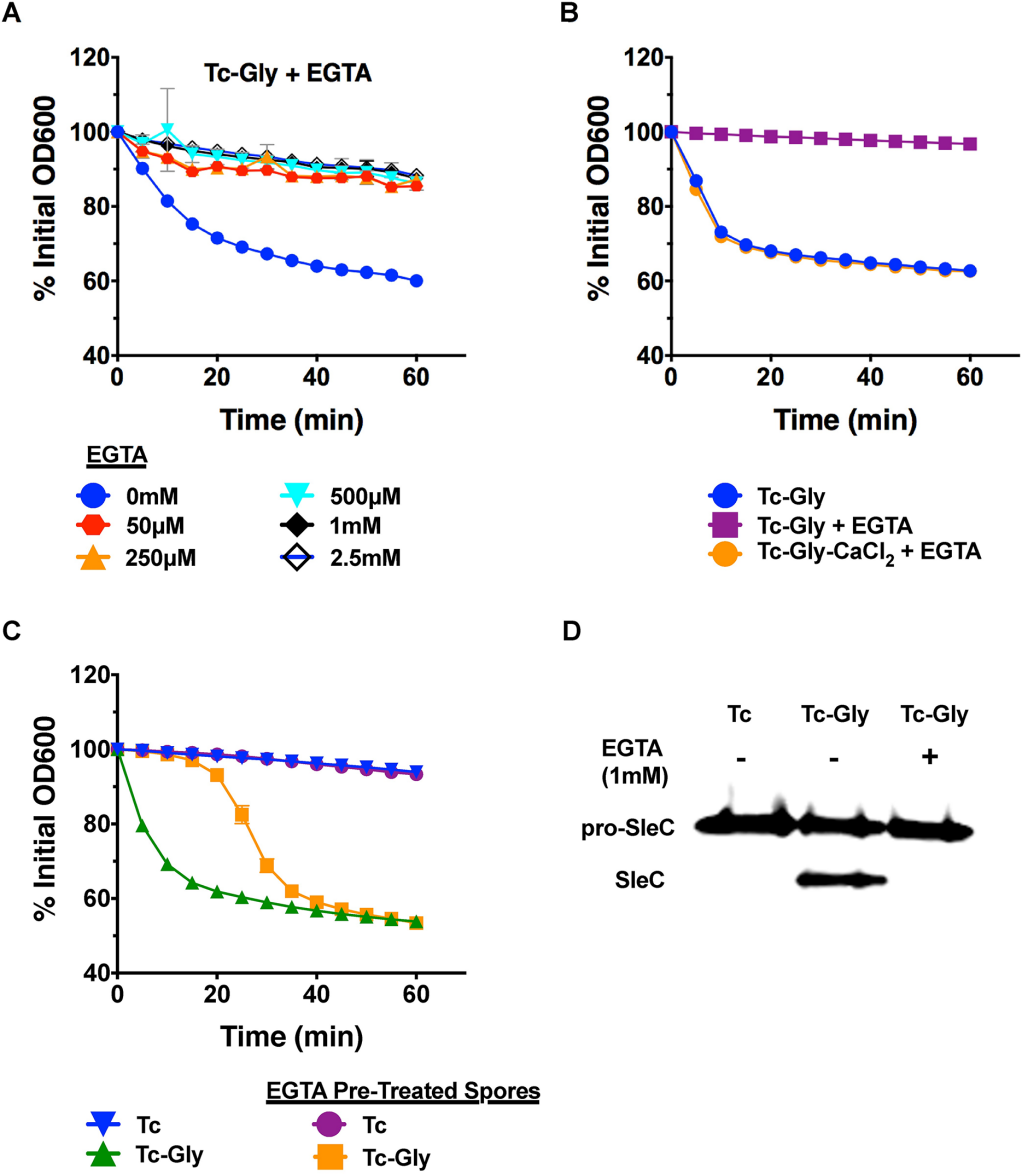
Calcium from within the spore is essential for germination in response to Tc-Gly. Cd630 spores were incubated with 0.2% Tc, 50mM glycine and different concentrations of EGTA (A). Cd630 spores were incubated with the indicated combinations of 0.2% Tc, 50 mM glycine, 1 mM CaCl_2_, and 50 μM EGTA (B). EGTA (1mM) pretreated Cd630 spores and non-treated Cd630 spores were incubated with the indicated combinations of chelex-treated germinants, Tc (0.2%) and glycine (50mM) (C). Activation of SleC was assessed by western blot. Cd630 spores were incubated 37°C for 15 minutes with 1% Tc and the indicated combinations of 50mM glycine and 1mM EGTA (D). Germination was tracked by loss of OD at 37°C over the course of one hour. Germination assays were performed in triplicate. Germination assays and western blots are representative of 3 independent spore preps. Error bars are mean plus or minus SD.

Since exogenous calcium is sufficient to induce germination (in the presence of Tc), we sought to understand the role of endogenous calcium in this process. To determine if endogenous calcium is required for Tc-Gly induced germination, Cd630 spores were incubated with Tc, glycine, and the calcium-specific chelator EGTA. EGTA inhibited Tc-Gly induced germination at all concentrations tested (Fig 2A), but was restored to wildtype levels with the addition of excess CaCl_2_ (Fig 2B). These data show that endogenous calcium is essential for *C*. *difficile* germination in the presence of the co-germinants, Tc-Gly. We hypothesize that calcium is functioning as an enzymatic cofactor for CspB and EGTA treatment will inhibit the activation of SleC. To test this hypothesis, Cd630 spores were incubated with Tc, Tc-Gly, or Tc-Gly-EGTA. As expected, no SleC processing was observed with Tc alone and SleC activation was observed in Tc-Gly treated spores (Fig 2D). However, EGTA completely inhibited SleC activation. These data support the hypothesis that calcium is functioning as signal to activate CspB, possibly by functioning as an enzymatic cofactor.

Since EGTA treatment inhibited Tc-Gly germination at concentrations as low as 50 μM, we hypothesized that spores may have Ca^2+^ in the spore coat/cortex layers. In order to test this hypothesis, we first chelex-treated Tc, glycine, and PBS to ensure there is no contaminating calcium, and then pretreated Cd630 spores with 1mM EGTA. These spores were washed 3 times with chelex-treated PBS and then incubated with chelex-treated Tc-Gly. EGTA pre-treated spores displayed ~20 min delay in germination as compared to untreated spores (Fig 2C). These data suggest that *C*. *difficile* spore outer layers contain small amounts of calcium (<50 μM) that when removed, delay germination until enough calcium is released from the spore core to activate SleC.

We hypothesized that endogenous calcium is transported out of the spore core and initiates cortex hydrolysis through the actions of calcium-dependent enzymes. In order to test this hypothesis, Cd630 spores were incubated with either 1mM Phenamil, an ion channel inhibitor [24], or 0.5mM Chlorpromazine (CPZ), an inhibitor of Ca^2+^-enzyme interactions[25] and either Tc-Gly or Tc-CaCl_2_. Phenamil-inhibited Tc-Gly-induced germination (~60% reduction) but not Tc-CaCl_2_-induced germination (>95% germination) (S2 Fig). These data suggest that Tc-CaCl_2_ induces germination independent of endogenous Ca^2+^ while Tc-Gly-induced germination requires Ca^2+^ efflux from the core. CPZ delayed both Tc-Gly and Tc-CaCl_2_ induced germination with an overall reduction of 30–50% (S2 Fig), indicating that calcium-dependent enzyme activation is required for efficient *C*. *difficile* germination. While the concentrations of CPZ and Phenamil are similar to that of other known spore inhibitors [24], we interpret these data cautiously given that they are higher than those used to inhibit eukaryotic cell targets. This difference in effective concentration could be due to low spore permeation.

### Tc-CaCl_2_ induces germination through a similar pathway as Tc-Gly

**Fig 3:**
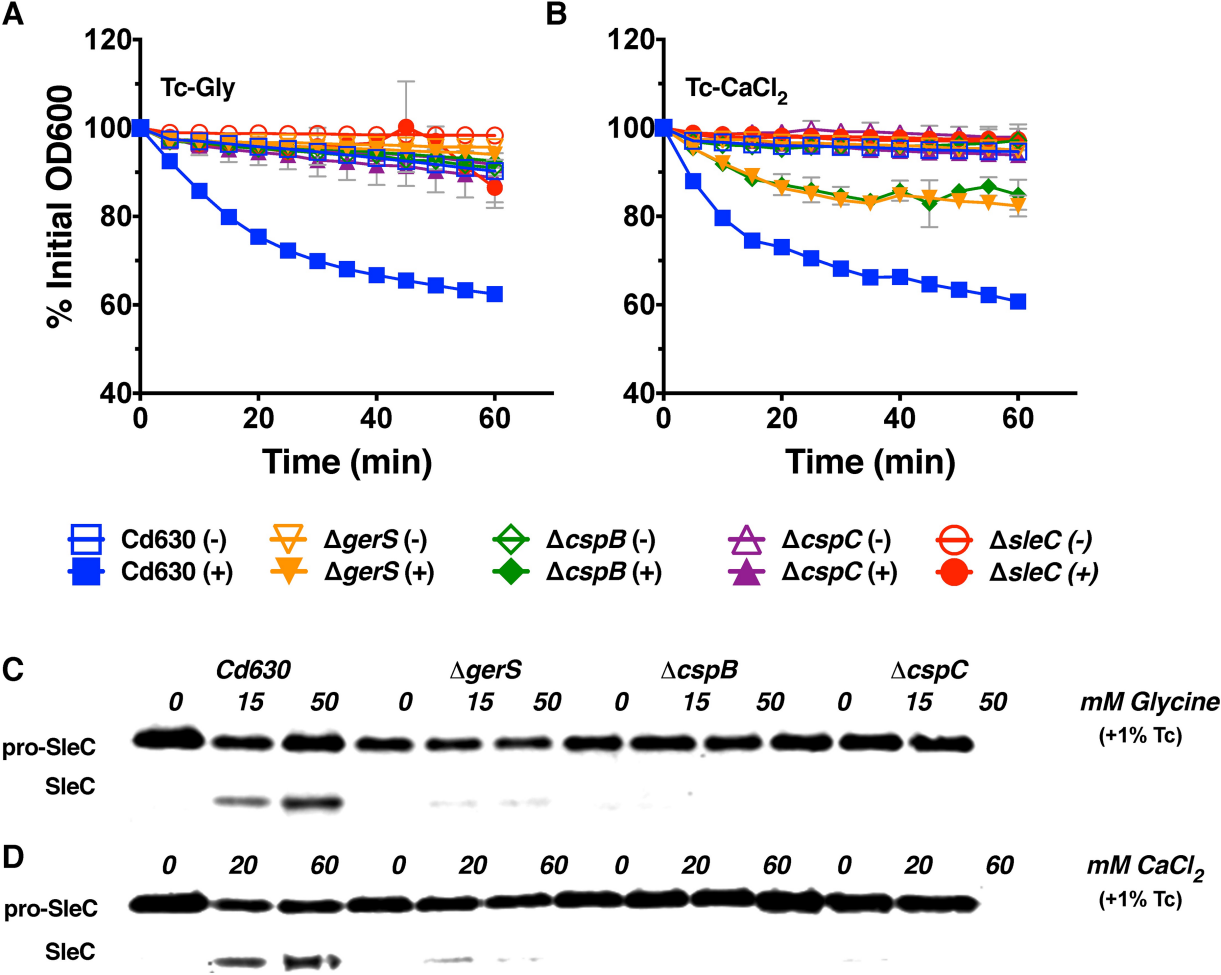
Tc-CaCl_2_ induced germination occurs through the same pathway as Tc-Gly. Cd630, Δ*gerS*, Δ*cspB*, Δ*cspC*, and Δ*sleC* spores were incubated with 0.2% Tc and with (+) or without (-) the indicated co-cogerminant: 50 mM glycine (a) or 60 mM CaCl_2_ (b). Germination was measured by loss of OD_600_ at 37°C over the course of one hour. (a-b). Western blot of SleC from WT, Δ*gerS*, Δ*cspB*, or Δ*cspC* spores incubated at 37°C for 15 minutes with 1% Tc and the indicated concentrations of glycine (c) or CaCl_2_ (d). Germination assays were performed in triplicate. Germination assays and western blots are representative of 3 independent spore preps. Error bars are mean plus or minus SD.

Since Tc-CaCl_2_ induced germination through the activation of SleC (Fig 1K), we hypothesized that this germinant combination signals through a similar pathway as Tc-Gly. To test this hypothesis, clean, unmarked deletions of genes essential for Tc-Gly-induced germination (*cspC*, *cspB*, *gerS*, *sleC*) were generated and germination kinetics were measured in response to Tc alone, Tc-Gly, or Tc-CaCl_2_ (Fig 3). None of the mutants germinated in response to Tc-Gly, confirming previously published reports of their importance for *C*. *difficile* germination (Fig 3A) [10,11,16]. In response to Tc-CaCl_2_, Δ*cspC* and Δ*sleC* spores did not germinate, but Δ*cspB* and Δ*gerS* spores exhibited low, but appreciable, levels of germination (~15% drop in OD_600_), possibly due to pro-SleC activity in the presence of high calcium (Fig 3B). As expected, zymogen processing of SleC was detected in Cd630 spores incubated with 1% Tc and all concentrations of either glycine or CaCl_2_ (Fig 3C and 3D). In contrast to a previous report (*14*), only minimal SleC activation was observed in Δ*gerS* spores incubated with either Tc-Gly or Tc-CaCl_2_ (Fig 3C and 3D). No zymogen processing was detected under any condition for Δ*cspB*, and Δ*cspC* spores (Fig 3C and 3D). These results indicate that both Tc-Gly and Tc-CaCl_2_ use the same set of enzymes in facilitating spore germination.

### Cd630_32980 is required for Tc-Gly but not Tc-CaCl_2_ induced germination

**Fig 4:**
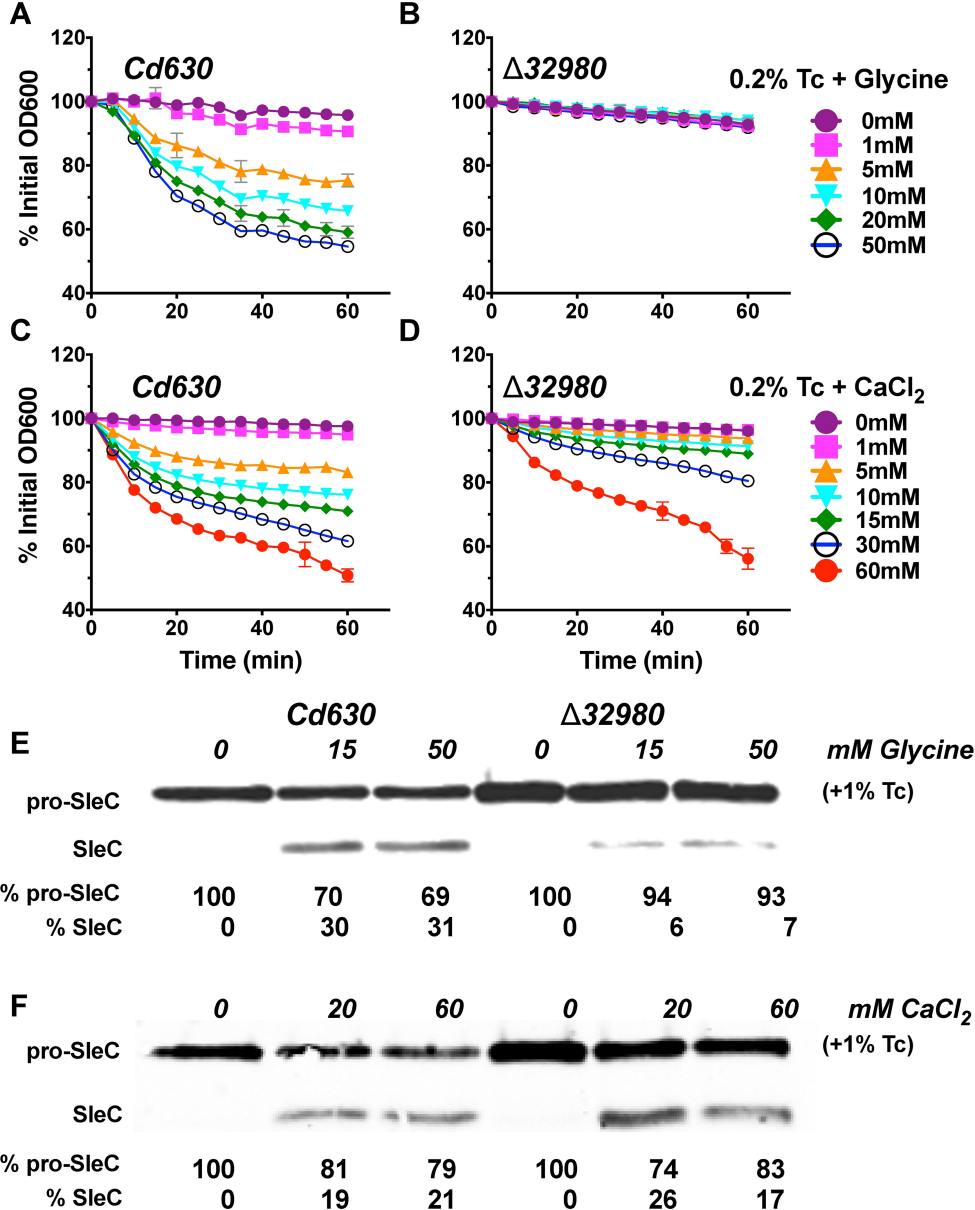
*Cd630_32980* is required for Tc-Gly induced germination. WT and Δ*32980* spores were incubated with 0.2% Tc and the indicated concentrations of glycine (a-b) or CaCl_2_ (c-d) with germination was measured by loss of OD_600_ at 37°C over the course of an hour. Western blot assessing SleC activation from WT and Δ*32980* spores incubated at 37°C for 15 minutes with 1% Tc and the indicated concentrations of glycine (e) or CaCl_2_ (f). %pro-SleC and % SleC relative densities were calculated for each lane using ImageJ by determining the ratio of each band to the total density for the two bands combined. Blots are representative of 3 experiments. Germination assays were performed in triplicate and are representative of 3 independent spore preps. Error bars are mean plus or minus SD.

Thus far we have shown that both Tc-Gly and Tc-CaCl_2_ induce germination through activation of SleC and that endogenous calcium transport is required for Tc-Gly germination but not Tc-CaCl_2_. We next sought to identify the step in the germination pathway where these mechanisms diverge. *Cd630_32980* is a gene identified as being highly expressed during sporulation [26] and predicted to be essential for *C*. *difficile* sporulation [27]. Our independent bioinformatics analysis of these data comparing the transposon depth of coverage of dormant spores to that of vegetative cells after germination suggested that this gene might also be essential for Tc-Gly germination. To test this hypothesis, we constructed a clean, unmarked deletion (Δ*32980*) and purified spores (albeit at a lower yield than Cd630). We next tested Δ*32980* spores for germination in 0.2% Tc with increasing concentrations of either glycine (0-50mM) or CaCl_2_ (0-60mM). Cd630 spores germinated in a dose-dependent manner in response to Tc-Gly (Fig 4A) but Δ*32980* spores did not germinate, regardless of the glycine concentration (Fig 4B). In contrast, Δ*32980* spores germinated in a dose-dependent manner in response to Tc-CaCl_2_, albeit at somewhat lower efficiencies than Cd630 at calcium concentrations ≤30mM (Fig 4C and 4D). Germination in Δ*32980* spores was restored by complementation of Cd630_32980 *in trans* (S3 Fig). Spores lacking Cd630_32980 exhibited minimal SleC activation in response to Tc-Gly, while retaining full Cd630 levels in response to Tc-CaCl_2_ (Fig 4E and 4F). These results suggest that Cd630_32980 is vital for Tc-Gly induced germination but not Tc-CaCl_2_.

*Cd630_32980* expression occurs in the mother cell and is controlled by the sporulation sigma factor SigE. *Cd630_32980* encodes a AAA+ ATPase that is associated with a putative type-4 secretion system predicted to be essential for sporulation [27]. We hypothesized that Cd630_32980 may be involved in transport of nutrients (including DPA) from the mother cell, across the outer forespore membrane into the spore during sporulation. To test this hypothesis, we measured the total amount of DPA that had been packaged into mature spores using a terbium fluorescence assay following a 30-minute boiling step to release internal stores of DPA. Δ*32980* spores contained <1% of the DPA content found in Cd630 spores but was rescued by expressing *Cd630_32980 in trans* (S3 Fig). These data indicate that Cd630_32980 is essential for the proper packaging of DPA in the spore. In addition, these data may explain why spore yields were low, as spores low in DPA are less dense and do not pellet readily during spore purification in 50% histodenz (see methods). Previous studies have demonstrated that Ca^2+^ and DPA are packaged in a 1:1 ratio and spores with less DPA have less internal stores of calcium [23,28]. To measure levels of calcium in mature spores we used a calcium colorimetric assay following a 30-minute boiling step to release internal stores of calcium. Δ*32980* spores contained ~5 μM calcium compared to ~250 μM calcium in either Cd630 or *Cd630_32980* complemented spores (S3 Fig). Taken with these data, our results suggest that DPA is essential for packaging of calcium into the *C*. *difficile* spore core and this calcium is essential for Tc-Gly germination.

### Calcium and glycine synergize with bile salts to induce *C*. *difficile* germination

Since Tc, glycine, and calcium are present within the host intestine we hypothesize that they function together to induce *C*. *difficile* germination. To test this hypothesis, we treated Cd630 spores with suboptimal concentrations of Tc (0.05%), glycine (5mM), or calcium (5mM). At these concentrations, Cd630 spores do not germinate in response to Tc-Gly or Tc-CaCl_2_. However, they germinated (~30% drop in OD) in response to the combination of Tc, glycine, and calcium (S4 Fig), indicating that glycine and calcium can synergize to induce *C*. *difficile* germination in the presence of Tc. Because complex growth media (e.g., BHIS, etc.) are commonly used as a germination media, and they typically contain both amino acids and calcium, we hypothesized that *C*. *difficile* germination in BHIS+Tc is due to the synergy between calcium and glycine. To test this hypothesis, we measured the concentration of calcium present in BHIS (0.4mM), calcium-depleted BHIS (CDP; 0mM), and calcium-replete BHIS (CRP; 1mM) (S5 Fig) prior to measuring Cd630 or *B*. *anthracis* Sterne 34F_2_ spore germination in each medium. Cd630 spores germinated in BHIS+Tc and CRP+Tc but did not germinate in CDP+Tc (S5 Fig). This indicates that the amino acid concentrations found in BHIS are insufficient for *C*. *difficile* germination in the absence of calcium. In addition, the concentration of calcium in BHIS (0.4mM) is insufficient to induce germination on its own (Fig 4C) suggesting that germination in BHIS+Tc is due to calcium-glycine synergy. In contrast, *B*. *anthracis* spores germinated fully in both BHIS and CDP (S5 Fig). These data indicate that *C*. *difficile* germination in BHIS is due to the combination of available amino acids and calcium while *B*. *anthracis* (which is not responsive to calcium, Fig 1H) germinates in response to amino acids and other nutrients available. Interestingly, Δ*32980* spores germinate slightly (~20% drop in OD) in BHIS+Tc but not in CDP+Tc (S5 Fig). This indicates that calcium present in BHIS is sufficient to induce slight germination in a strain that is lacking DPA as also reported by Donney et al. [22]. In accordance with these data, we propose that within the host intestine, (where Tc, glycine, and calcium are all present) these stimuli function to decrease the concentrations of individual germinants required for *C*. *difficile* germination and thus colonization within the host.

### Intestinal calcium plays a key role during *in vivo* germination in a murine model of CDI

**Fig 5:**
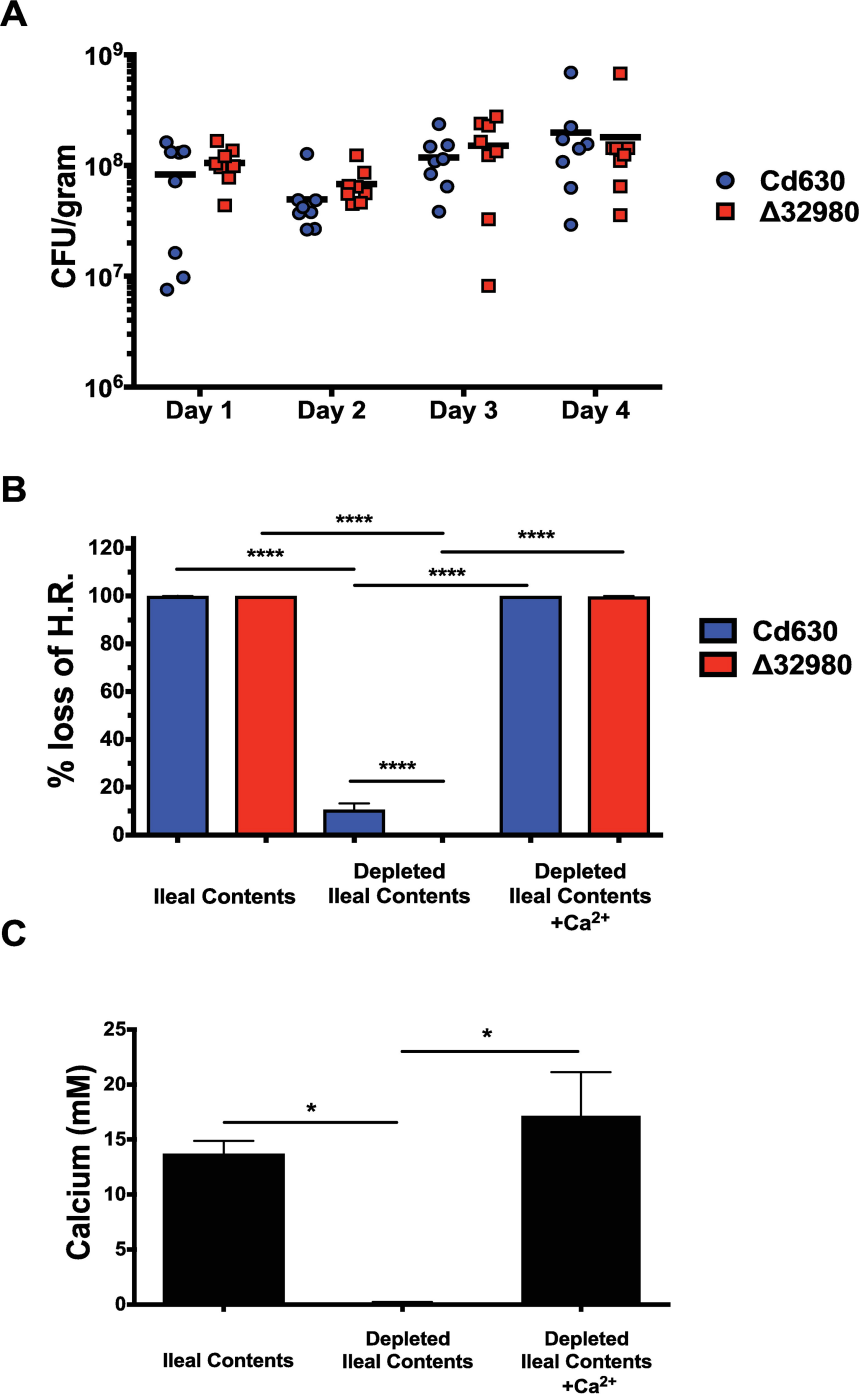
Tc-Gly induced germination is dispensable for *in vivo* germination of *C*. *difficile* spores. Cefoperazone-treated C57BL/6 mice (n = 8) were infected with 1500 spores of WT or Δ*32980* by oral gavage. Colonization levels were assessed daily and are presented in total CFU per gram of feces. (a) Multiple t tests were performed and p = 0.23 for each time point tested (a). Ileal contents were collected from Cefoperazone treated C57BL/6 mice (n = 3) and *ex vivo* germination assays were performed. 1x10^3^ spores of Cd630 or Δ*32980* were incubated for one hour at 37°C in ileal contents, calcium depleted ileal contents or calcium depleted ileal contents treated with 15 mM CaCl_2_. Samples were then incubated at 65°C for 20 min and then plated on BHIS-Tc plates. Data are presented as % loss of Heat Resistance (b). Free calcium levels of ileal contents, calcium depleted ileal contents or calcium depleted ileal contents treated with 15 mM CaCl_2_ were measured using a calcium colorimetric assay. Levels of calcium (mM) were determined using a standard curve (c). Assays were performed in triplicate using ileal contents from three mice. Error bars are mean plus or minus SD. Statistical significance was calculated using Two-way ANOVA. (*) p<0.05 (****) p<0.0001.

Since bile salts, glycine, and calcium each play a role in *C*. *difficile* germination and synergize to increase germination levels at low concentrations, we hypothesize that dietary calcium (800–1300 mg/day)[29] coordinates with Tc (0.03%) [30], in the host intestines. In order to test this hypothesis, mice were pre-sensitized to *C*. *difficile* colonization with antibiotic therapy [31] (see methods) and inoculated with either Cd630 or Δ*32980* spores (which is deficient for endogenous calcium). Mice infected with either strain exhibited similar levels of *C*. *difficile* in the stool (Fig 5A) indicating that Cd630_32980, endogenous calcium, and therefore Tc-Gly-induced germination, are not essential for *in vivo* germination in our murine model. These data, in combination with our *in vitro* findings, suggest that intestinal calcium plays a role in *C*. *difficile* germination bypassing the requirement for glycine. To directly test if intestinal calcium plays a role in *C*. *difficile* spore germination, ileal contents were collected from antibiotic-treated, non-infected mice, calcium was depleted, and *ex vivo* germination assays were performed. In mouse ileal contents, that were found to contain ~15mM calcium, 100% of both Cd630 and Δ*32980* spores germinated within one hour (Fig 5B). However, when calcium levels were depleted using chelex resin (Fig 5C), only 10% of WT and no Δ*32980* spores germinated (Fig 5B). Complete germination was restored through the addition of supplemental calcium (~15mM), for both Cd630 and Δ*32980* spores (Fig 5B). These data indicate that calcium in the intestines is required for efficient *C*. *difficile* spore germination. Taken together, these data show that intestinal calcium is a key molecule involved in *C*. *difficile* germination and imply that modulation of intestinal calcium represents a means to decrease germination, colonization, and pathogenesis.

## Discussion

**Fig 6:**
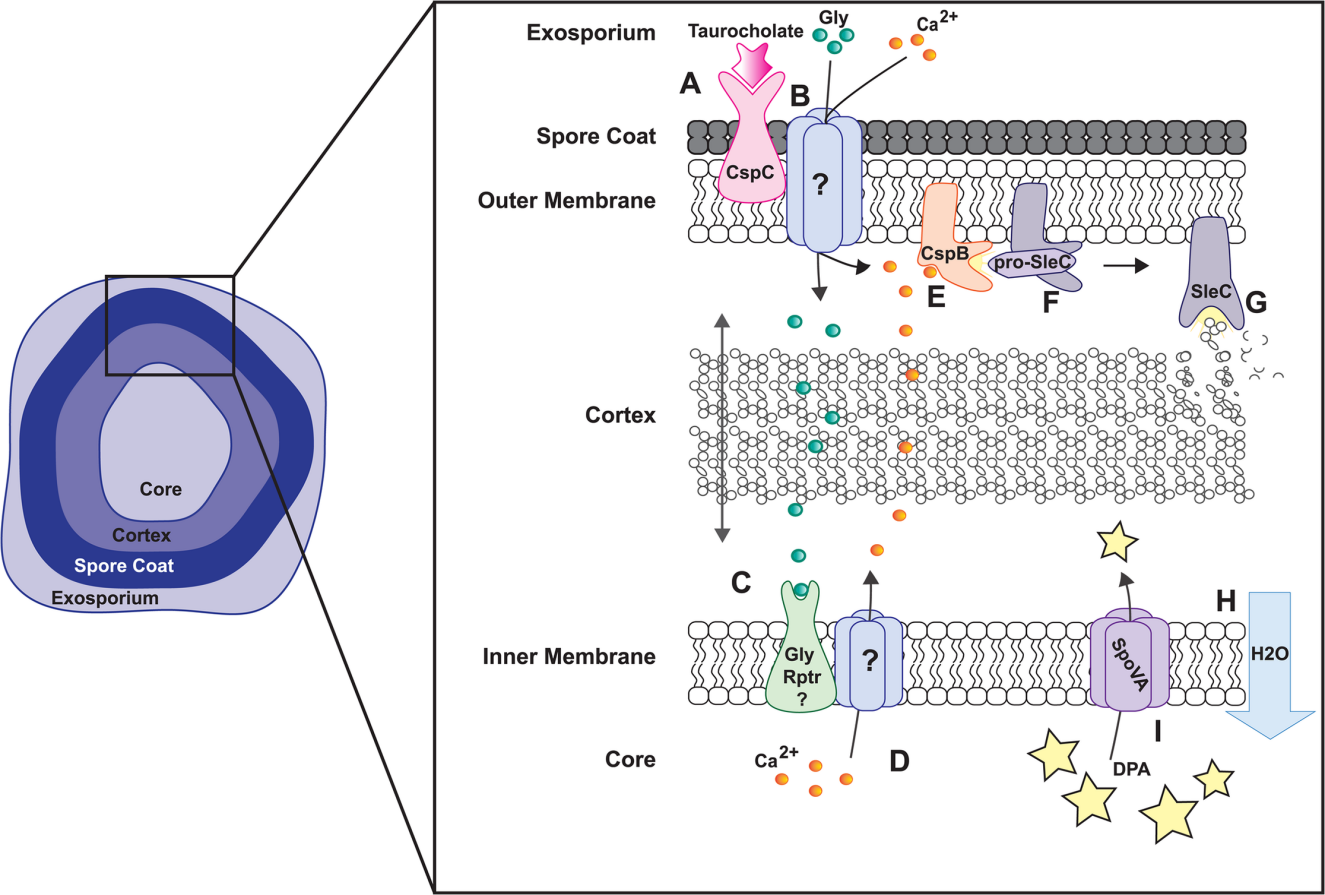
Proposed model for the role of calcium in *C*. *difficile* germination. Tc binds to CspC (A) facilitating movement of glycine or calcium through the spore coat and outer membrane (B). Glycine then interacts with an unknown receptor (C) inducing the release of Ca^2+^ from the spore core (D). Ca^2+^ from the environment or the spore core activates CspB (E), which processes pro-SleC (F) subsequently initiating cortex hydrolysis (G). This leads to full core rehydration (H), complete release of DPA (I) and spore outgrowth.

The unconventional mechanism of *C*. *difficile* spore germination has remained elusive due to the absence of known germinant receptor orthologues. In this work, we describe a central role for calcium ions in *C*. *difficile* germination. Our data shows that *C*. *difficile* spores germinate in response to a combination of bile salts and intestinal calcium. Amino acid concentrations within the mouse gastrointestinal tract are inadequate to support high levels of germination independent of intestinal calcium. This is the first report of intestinal calcium playing a vital role in *C*. *difficile* spore germination. We also provide evidence that endogenous calcium ions released from the spore core in response to Tc-Gly can serve as a germination signal by activating cortex hydrolysis. The putative AAA+ ATPase encoded by *Cd630_32980* is essential for proper packaging of DPA during sporulation as well as germination in response to Tc-Gly (but not Tc-CaCl_2_). Despite the lack of glycine-induced germination, Δ*32980* spores retain the ability to germinate in *ex vivo* ileal contents and colonize the mouse gastrointestinal tract to levels identical to that of Cd630. In calcium-depleted ileal contents, no mutant spores germinated and Cd630 spores had a 90% reduction in germination. Our data supports the hypothesis that *C*. *difficile* germination *in vivo* occurs due to synergistic effects between bile salts, glycine, and calcium. The data presented here suggest that the role of glycine in *C*. *difficile* germination is to facilitate calcium release from the spore core (Fig 6) and that this mechanism can be circumvented with the addition of exogenous calcium. In short, *C*. *difficile* spore germination requires calcium that can be provided by either environmental or endogenous sources. Collectively, these data suggest that restricting free intestinal calcium in susceptible patients is a potential prophylactic treatment to inhibit human CDI.

While recent studies have begun to elucidate *C*. *difficile* germination, the mechanisms responsible for initiating these critical events are not completely understood. The subtilisin-like CspBAC family of proteases, a novel lipoprotein regulator GerS, and the cortex lytic enzyme SleC have been identified as key regulators in *C*. *difficile* germination [10,11,15,16]. Each of these proteins are essential for germination in response to Tc-Gly and, as described here, Tc-CaCl_2_. The identical role for these proteins in both germination mechanisms indicates that Tc-CaCl_2_ triggers germination upstream of CspB and downstream of the unidentified glycine receptor (Fig 6B). In our proposed model, Tc binds to CspC, (Fig 6A) allowing environmental calcium and/or amino acids to penetrate the spore coat (Fig 6B). While there are two hypotheses for the function of CspC—directly activating CspB or facilitating germinant entry into the spore—we prefer the latter explanation but do not want to discount the possibility that CspC functions to activate CspB. Our data support the hypothesis that glycine interacts with an unknown receptor (Fig 6C) and induces the release of calcium ions from the spore core (Fig 6D). Calcium released from within the spore, and/or calcium from the environment, then activates the subtilisin protease, CspB (Fig 6E), which induces SleC zymogen processing and activation (Fig 6F). Activated SleC then hydrolyzes the spore cortex, leading to full-core rehydration, DPA release through SpoVA channels [22], and spore outgrowth (Fig 6H and 6I).

Since exogenous CaCl_2_ initiates cortex hydrolysis through CspB and SleC activation (Figs 1K & 3D), and EGTA treatment inhibits SleC activation (Fig 2D), we hypothesize that calcium is a necessary cofactor for CspB activity. Calcium and the activity of calcium dependent enzymes are required for *C*. *difficile* germination as demonstrated by EGTA calcium chelation and CPZ treatment studies (Figs 2A–2C, S2C and S2D) demonstrating a requirement for calcium. EGTA pre-treatment and Phenamil treatment studies indicate that calcium efflux from the spore core is required for Tc-Gly induced germination (S2A and S2B Fig). Exogenous Tc-calcium induced germination at a maximal rate of 5.2% germination/min and the concentration of calcium required to reach half of the maximal germination rate is 12.6mM (extrapolated [32] from Fig 4C). The concentration of exogenous calcium required to induce germination then is much higher than the concentration of EGTA (50 μM) needed to chelate endogenous calcium and inhibit Tc-Gly germination. This discrepancy could be partially explained by low permeability of the spore coat to exogenous calcium thus, greater concentrations of exogenous calcium are required. We also speculate that endogenous calcium, following release from the spore core, could participate in a positive feedback loop inducing release of more calcium until a threshold concentration is achieved and CspB is activated. Very low concentrations of EGTA could interrupt this positive feedback loop. These data support our model that calcium is vital for SleC activation and thus *C*. *difficile* germination.

Clinical and epidemiological studies have demonstrated a correlation between risk of CDI and patients who have defects in calcium absorption. For example, patients taking proton pump inhibitors (PPIs) have an elevated risk of contracting CDI [33–36]. PPIs are commonly prescribed to ICU patients for numerous reasons (*e*.*g*., peptic ulcer diseases, upper gastrointestinal bleeding, stress ulcer prophylaxis), many of these patients may also be on antibiotics. A known side effect of this treatment is decreased calcium absorption in the small intestine resulting in increased luminal concentrations of calcium [37,38]. In light of our results, it is possible that elevated levels of intestinal Ca^2+^ in these individuals allows for more efficient germination of *C*. *difficile* in the small intestines and therefore increased risk of infection. Additionally, a potential correlation between age, dietary calcium, and CDI exists as elderly individuals exhibit decreased absorption of calcium and have a significantly higher incidence and severity of CDI. Finally, patients deficient in vitamin D, which is required for calcium absorption from the gut, are five times more likely to contract CDI [39,40]. While the classical role of vitamin D is to facilitate calcium absorption in the small intestines [41,42], it also plays a role in innate immunity and the regulation of mucosal immunity [43,44]. A deficiency in vitamin D could increase the risk of *C*. *difficile* colonization through elevated levels of available germinant (calcium) in the intestines and an inefficient immune response leading to severe disease.

The *C*. *difficile* germination mechanism presented here, together with clinical correlations regarding calcium absorption, suggests an exploitable target for the development of new therapeutic strategies. Decreasing intestinal calcium levels in at-risk patients (*e*.*g*., by vitamin D supplementation to improve calcium absorption, [40]) represents a novel prophylactic approach for influencing establishment, outcome, or recurrence of CDIs. In contrast, increasing intestinal calcium levels during CDI treatment could facilitate germination of resident spores, rendering them susceptible to antibiotic treatment, and thus decreasing both spore dissemination and incidence of recurrent CDI. In light of our findings, future studies are warranted to determine the clinical benefits of modulating intestinal calcium levels to improve CDI outcomes.

## Materials and methods

### Bacterial strains and growth conditions

*C*. *difficile* strains used in this study are described in S1 Table *C*. *difficile* was grown in an anaerobic chamber (10% hydrogen, 5% CO_2_, 85% N_2_) (Coy Lab Products, MI) at 37°C in brain-heart infusion broth (BD Life Sciences) supplemented with 0.5% yeast extract (BD Life Sciences) and 0.1% cysteine (Sigma-Aldrich) (BHIS). *Escherichia coli* strains were grown at 37°C in Luria-Bertani (LB) broth (BD Life Sciences) or LB agar (Fisher Scientific) supplemented with the appropriate antibiotics. All antibiotics were purchased from Sigma-Aldrich and used at the following concentrations: for *C*. *difficile*, thiamphenicol (15 μg/mL); for *E*. *coli*, ampicillin (50 μg/mL), chloramphenicol (25 μg/mL). Conjugations are plated on BHIS plates supplemented with cefoxitin (8μg/mL), D-cycloserine (250 μg/mL), and thiamphenicol (*15* μg/mL) (CCT). Secondary crossovers were selected by plating on chemically defined media plates (CDMM) supplemented with fluorocytosine (50 μg/mL) [45].

### Spore production and purification

Spores were generated as follows: *C*. *difficile* was allowed to grow overnight at 30°C in Columbia broth (BD Life Sciences) and 2mL of culture were added to 38 mL of Clospore sporulation media [46] then incubated at 37°C for 6 days. Spores were collected by centrifugation at 4,000 RPM and washed 3 times with sterile distilled, deionized water (ddH_2_O, Millipore). To remove vegetative cells and debris, spore pellets were re-suspended in 50% Histodenz (Sigma-Aldrich) and centrifuged at 13,200 RPM [10]. Supernatant was discarded and spore pellets washed 3 times with sterile ddH_2_0. Spore purity was >95% as confirmed by phase contrast microscopy.

### Cloning and construction of *C*. *difficile* mutants

Clean unmarked deletions in *C*. *difficile* were created using a protocol modified from *Cartman et al*. [45]. Briefly, 1,000 bp fragments of DNA flanking the target gene were Gibson cloned into the NotI site of plasmid pMTL-SC7215 (Gibson Assembly Master Mix, New England Biosciences). Plasmids were conjugated into Cd630 via the *E*. *coli* strain HB101 which harbors the conjugative plasmid pRk24 [45]. Conjugations were plated on BHIS for 24 hours to allow transfer of the plasmid. Bacterial growth was scraped off and plated on CCT for 2 days and colonies were picked and re-streaked for isolation. Primary insertions were confirmed via PCR using primers described in S2 Table. A pure culture of an isolate with a confirmed primary insertion was then plated on BHIS without selection overnight to allow for secondary crossover events to occur. Secondary crossovers were selected by plating on CDMM supplemented with fluorocytosine, selecting against colonies carrying the original knockout plasmid. Single colonies were picked and screened for deletion of targeted genes by PCR.

### Loss of OD germination assay

Germination was measured by tracking the loss of optical density at 600 nm over time at 37° C in a Spectramax M2 microplate reader (Molecular Devices). Loss of OD following full rehydration of the core is a known indicator of spore germination [11,21,47]. Purified spores were added to phosphate buffer saline (PBS, Invitrogen) with the indicated germinants at a starting OD of ~0.5. For Ca-DPA or CaCl_2_ induced germination, spores were added to 50 mM Tris-HCl to a pH of 7.4 (Sigma-Aldrich) plus the indicated germinants. The OD_600_ was taken every 5 minutes for one hour with the results reported as percent initial OD_600_. Assays were performed in triplicate.

### Germination inhibitor studies

Germination inhibitors, (Phenamil, EGTA, or Chlorpromazine) were purchased from Sigma-Aldrich. EGTA was solubilized in 100 mM Tris-HCl, pH 7.4. Phenamil was re-suspended in 100% DMSO. All germination assays in which Phenamil was used were conducted in 10% DMSO. Chlorpromazine is freely soluble in water.

### Loss of heat resistance assay

Germination was also measured by loss of heat resistance after one hour. 1x10^3^ Cd630 spores were incubated with 0.2% Tc (Sigma-Aldrich) and 50 mM glycine (Sigma-Aldrich) or 60 mM CaCl_2_ (Sigma-Aldrich) at 37°C. After one hour, samples were heat treated at 65°C for 20 minutes, serially diluted in PBS, and plated on BHIS+Tc plates. Non-heat-treated samples (total spores) were also plated on BHIS+Tc. Data are reported as a percentage of the total spores that lost the heat resistance properties of dormant spores.

### Western blot analysis

Zymogen processing of SleC was detected by western blot using an anti-SleC antibody [16] graciously provided by Dr. Aimee Shen, Tufts University. Here, 1x10^6^ spores were added to the indicated germination mixture and incubated at 37°C for 15 minutes. Spores were then pelleted and re-suspended in 100 μL EBB lysis buffer (9M urea, 2M thiourea, 4% SDS, and 10% β-mercaptoethanol) prior to the addition of 10 μL of 4x loading buffer to the protein lysates[16]. Proteins were separated on a 4–12% SDS-PAGE gel and transferred to a 0.22 μm nitrocellulose membrane (Whatman). Membranes were blocked for one hour in Odyssey blocking buffer (LI-COR) then probed for one hour at room temperature with an anti-SleC antibody (1:5000). Membranes were washed 3 times for 10 minutes in TBS-T. Goat anti-rabbit IR800 secondary antibodies (LI-COR) were added at a 1:20,000 dilution and incubated at room temperature for an hour. The membranes were washed a minimum of 3 times with TBS-T before LICOR detection (Odyssey).

### Monitoring DPA release assay

DPA release from the spore core was measured using terbium fluorescence [21]. Cd630 spores were incubated with 0.2% Tc and 50mM glycine in PBS or 60mM CaCl_2_ in Tris-HCl at 37°C for 1 hour. Germinant solutions were supplemented with 800μM TbCl_3_ (Sigma Aldrich) to measure Ca-DPA release in real-time using a spectramax M2 microplate reader (Molecular Devices) (excitation 270 nm, emission 545 nm, cutoff 420 nm). Data is presented as relative fluorescent units. For measuring total amounts of DPA packaged into spores, 1x10^8^ Cd630 spores were incubated at 100°C for 30 minutes. Boiled samples were supplemented with 800μM TbCl_3_ and DPA release was measured.

### Murine model of *Clostridium difficile* colitis

8-week old C57BL/6 mice were given cefoperazone (Sigma-Aldrich) (0.5 mg/ml) in sterile drinking water for five days which was refreshed every other day [31]. Mice were then switched to regular drinking water, allowed to recover for 2 days prior to *C*. *difficile* infection. For *ex vivo* germination assays, uninfected mice (n = 3) were euthanized, and at the time of necropsy ileal contents were collected, and frozen at -80°C until further analysis. For *in vivo* infections, groups of mice (n = 8) were inoculated by oral gavage with 50μL of water containing approximately 1500 spores as determined by phase contrast microscopy. Feces were collected daily for 4 days and samples weighed, serially diluted, and plated for total CFU per gram of feces (spores + vegetative cells). Samples from day one were also heat treated and assayed for total heat resistant CFU (spores). Mice were placed on a standard diet of Prolab Isopro RMH 3000 (LabDiet, St. Louis, MO) containing 1.1% calcium. C57BL/6 mice consume an average of ~4 g of chow per day [48].

### *Ex vivo* germination assays

Mouse ileal contents from uninfected antibiotic treated mice were weighed and diluted 1:1 in PBS. Samples were then freeze-thawed three times to release any available nutrients and centrifuged at 13,200 RPMs for 2 minutes and supernatants were collected. ~1x10^3^ Cd630 or Δ*32980* spores were added to PBS, diluted ileal contents, calcium depleted ileal contents, or calcium replete ileal contents and incubated at 37°C. After one hour, samples were heat treated at 65°C for 20 minutes, serially diluted in PBS, and plated on BHIS+Tc plates. Non-heat-treated samples (total spores) were also plated on BHIS+Tc. Data are reported as a percentage of the total spores that lost the heat resistance properties of dormant spores.

### Calcium depletion

Calcium was depleted by incubating BHIS or ileal contents for 2 hours with 0.1g/mL chelex 100 resin (BioRad) and then removing the resin by centrifugation for 2 minutes at 13,200 RPMs and collecting the supernatant. Calcium-replete BHIS was made by adding 1mM CaCl_2_ to calcium-depleted BHIS. Calcium-replete ileal contents were made by adding 15mM CaCl_2_ to calcium-depleted ileal contents.

### Calcium colorimetric assay

Calcium levels were measured using a calcium colorimetric assay purchased from Sigma-Aldrich. Ileal contents were diluted 1:200 in PBS to fit into the linear range of the assay. Briefly, 90μL of the chromogenic reagent was added to each well. 50μL of either sample or diluted calcium standard were added to each well. 60μL of calcium assay buffer are then added to each well and samples were incubated for 5–10 minutes at room temperature and absorbance was measured at 575nm in a Spectramax M2 microplate reader (Molecular Devices). Each sample was measured in triplicate.

### Ethics statement

*C*. *difficile* mouse infections were performed at the US Food & Drug administration. All animal procedures were approved by the CBER Animal Care and Use Committee (Protocol #2015–08) in accordance with the principles outlined in the Guide for the Care and Use of Laboratory Animals by the Institute for Laboratory Animal Resources, National Research Council. All experiments were performed in an Association for Assessment and Accreditation of Laboratory Animal Care International approved facility. We have calculated that 8 mice per group are required for power analysis (assuming 80% power) for the desired P value of 0.05, a standard deviation of <10% and a failure rate of <10%.

## Supporting information

S1 Fig Cation induction of spore germination. Cd630 spores were incubated with 0.2% Tc and 60 mM of the indicated cation (A). *Bacillus anthracis* (Sterne 34F_2_) spores were incubated with 60 mM DPA and 60 mM of the indicated cation (B). Germination was measured by tracking loss of optical density at 600 nm at 37°C over the course of an hour. Germination assays were performed in triplicate and are representative of 3 independent spore preps. Error bars are mean plus or minus SD.
(EPS)

S2 Fig Chemical inhibitors of spore germination. Cd630 spores were incubated with the indicated combinations of 0.2% Tc, 50 mM glycine, 60mM CaCl_2_, and 1 mM Phenamil (A,B) or 0.5 mM CPZ (C,D). Germination was measured by tracking loss of optical density at 600 nm at 37°C over the course of an hour. Germination assays were performed in triplicate and are representative of 3 independent spore preps. Error bars are mean plus or minus SD.
(EPS)

S3 Fig Complementation of Cd630:Δ*32980*. Cd630, Δ*32980*, or Δ*32980*/*32980*^+^ spores were incubated at 37°C with 0.2% Tc and 50 mM Glycine (A). Germination was tracked by loss of optical density. Time-points were taken every 5 minutes for one hour. Cd630, Δ*32980*, or Δ*32980*/*32980*^+^ spores were incubated at 100°C for 20 minutes and total DPA content was measured using Terbium fluorescence and calcium content was measured using a colorimetric assay (B). DPA release units are displayed as relative fluorescent units. Calcium concentrations were determined using a standard curve. Germination assays were performed in triplicate and are representative of 3 independent spore preps. Error bars are mean plus or minus SD. Statistical significance was calculated using one-way ANOVA (****) p<0.0001 (***) p<0.001.
(EPS)

S4 Fig Calcium and glycine synergize with bile salts to induce *C*. *difficile* germination. Cd630 spores were incubated at 37°C with suboptimal concentrations of Tc (0.05%), Glycine (5 mM), or Calcium (5 mM). Germination was tracked by loss of optical density. Time-points were taken every 5 minutes for one hour. Germination assays were performed in triplicate and are representative of 3 independent spore preps. Error bars are mean plus or minus SD.
(EPS)

S5 Fig Germination in BHIS is due to Calcium/Glycine synergy. Sterne 34F2 spores were incubated in BHIS or Calcium Deplete BHIS (CDP) (A). Cd630 spores were incubated in BHIS, CDP, or CRP supplemented with 0.2% Tc (A, B). Calcium concentrations of BHIS, CDP, or Calcium Replete BHIS (CRP) were measured using a calcium colorimetric assay. Calcium concentrations were calculated using a calcium standard curve (C). Δ*32980* spores were incubated at 37°C in BHIS, CDP, or CRP supplemented with 0.2% Tc (D). Germination was measured by loss of optical density. Time-points were taken at 37°C every 5 minutes for one hour (A, B, D). Germination assays were performed in triplicate and are representative of 3 independent spore preps. Error bars are mean plus or minus SD.
(EPS)

S1 Table Strains and plasmids used in this study. (DOCX)

S2 Table Primers used in this study. (DOCX)
