# Correction: Intestinal calcium and bile salts facilitate germination of *Clostridium difficile* spores

**DOI:** 10.1371/journal.ppat.1006605

**Published:** 2017-09-07

**Authors:** The PLOS Pathogens Staff

There is an error in the references. Reference 48 should be listed as reference 20. As a result of this error, the reference list is out of order. Reference 20 in the list should be reference 21, reference 21 should be reference 22, reference 22 should be reference 23, and so on, as far as reference 47, which should be reference 48. The publisher apologizes for the error.
