# Supplementary material for: Intestinal calcium and bile salts facilitate germination of Clostridium difficile spores
Source: PLoS Pathog. 2017 Jul 13;13(7):e1006443. doi: 10.1371/journal.ppat.1006443 (PMC5509370; doi:10.1371/journal.ppat.1006443)
Supplement: S1 Table — (DOCX) [file ppat.1006443.s006.docx]

**S1 Table**

| Strain # | Strain | | Relevant Genotype | Source |
| --- | --- | --- | --- | --- |
| *C. difficile* strains | |  |  |  |
|  | Cd630 | |  | ATCC BAA-1382 |
|  | R20291 | |  | ([1](#_ENREF_1)) |
|  | VPI 10463 | |  | ATCC 43255 |
| 94 | Δ*cspC* | | Cd630*:Δ22460* | This Study |
| 121 | Δ*cspB* | | Cd630*:Δ13550* | This Study |
| 85 | *ΔgerS* | | Cd630*:Δ34640* | This Study |
| 107 | *ΔsleC* | | Cd630*:Δ05510* | This Study |
| 97 | *Δ32980* | | Cd630*:Δ32980* | This Study |
| 144 | *Δ32980:32980^+^* | | Cd630*:Δ32980 +* pMTL-83151+32980^+^ Thi^R^ | This Study |
|  |  | |  |  |
| *E.coli* strains |  | |  |  |
| SD46 | HB101 | | pRk24 Amp^R^ | ([2](#_ENREF_2)) |
| 84 | HB101 | | pMTL-SC7215-cspC Cam^R^ pRk24 Amp^R^ | This Study |
| 120 | HB101 | | pMTL-SC7215-cspB Cam^R^ pRk24 Amp^R^ | This Study |
| 106 | HB101 | | pMTL-SC7215-sleC Cam^R^ pRk24 Amp^R^ | This Study |
| 71 | HB101 | | pMTL-SC7215-gerS Cam^R^ pRk24 Amp^R^ | This Study |
| 152 | HB101 | | pMTL-SC7215-32980 Cam^R^ pRk24 Amp^R^ | This Study |
| 148 | HB101 | | pMTL-83151+32980^+^32980 Cam^R^ pRk24 Amp^R^ | This Study |
| *B. anthracis* strains |  | |  |  |
|  | Sterne 34F2 | |  | ([3](#_ENREF_3)) |
|  |  | |  |  |
| Plasmids |  | |  |  |
|  | pMTL-SC7215 | | Cam^R^ | ([2](#_ENREF_2)) |
|  | pMTL-83151 | | Cam^R^ | ([2](#_ENREF_2)) |
|  | pMTL-83151+32980^+^ | | Cam^R^ | This Study |
|  |  | |  |  |

1. Stabler RA, He M, Dawson L, Martin M, Valiente E, Corton C, et al. Comparative genome and phenotypic analysis of Clostridium difficile 027 strains provides insight into the evolution of a hypervirulent bacterium. Genome biology. 2009;10(9):R102. PubMed PMID: 19781061. Pubmed Central PMCID: 2768977.

2. Cartman ST, Kelly ML, Heeg D, Heap JT, Minton NP. Precise manipulation of the Clostridium difficile chromosome reveals a lack of association between the tcdC genotype and toxin production. Applied and environmental microbiology. 2012 Jul;78(13):4683-90. PubMed PMID: 22522680. Pubmed Central PMCID: 3370502.

3. Wilson MJ, Carlson PE, Janes BK, Hanna PC. Membrane topology of the Bacillus anthracis GerH germinant receptor proteins. Journal of bacteriology. 2012 Mar;194(6):1369-77. PubMed PMID: 22178966. Pubmed Central PMCID: 3294866.
