# Supplementary material for: Intestinal calcium and bile salts facilitate germination of Clostridium difficile spores
Source: PLoS Pathog. 2017 Jul 13;13(7):e1006443. doi: 10.1371/journal.ppat.1006443 (PMC5509370; doi:10.1371/journal.ppat.1006443)
Supplement: S2 Table — (DOCX) [file ppat.1006443.s007.docx]

**S2. Table**

| Primer | Sequence | Description |
| --- | --- | --- |
| 1 | AATTTTTTTATCAGGAAACAGCTATGACCGCCCTATGTACTGTTCTTTTTCTCTG | P1 Forward Primer to amplify 1000bp upstream cspC |
| 2 | GGATTTATGGAAAAAACTCTATAGATAAGAACCTATGTAA | P2 Reverse Primer to amplify 1000bp upstream cspC |
| 3 | TCTTATCTATAGAGTTTTTTCCATAAATCCCTCCTATCTT | P3 Forward Primer to amplify 1000bp downstream cspC |
| 4 | GTAATCATGGTCATATGGATACAGCGGCC TGCATCTCAGTATGCATATAAAAAA | P4 Reverse Primer to amplify 1000bp downstream cspC |
| 5 | AAAAGTATTACTTCTTGGTCTTAAATGTGCTATCGTT | P5 Forward primer to amplify cspC |
| 6 | ATAATAAAACTCGGCAAGATAGATGATTTTTATAATAGTG | P6 Reverse primer to amplify cspC |
| 7 | AATTTTTTTATCAGGAAACAGCTATGACCG TCTTGATAAATTAAGAGCTAAATTG | P1 Forward Primer to amplify 1000bp upstream cspB |
| 8 | TCCCTCCTATCTTAATAAAATGTATTAGACTATATAATTT | P2 Reverse Primer to amplify 1000bp upstream cspB |
| 9 | GTCTAATACATTTTATTAAGATAGGAGGGATTTATGGAAA | P3 Forward Primer to amplify 1000bp downstream cspB |
| 10 | GTAATCATGGTCATATGGATACAGCGGCC AACTTCACCTGAAGGAGATATTATC | P4 Reverse Primer to amplify 1000bp downstream cspB |
| 11 | AAGACACTTATAGTTAATAGGAGCTGGATAATTATGTT | P5 Forward primer to amplify cspB |
| 12 | TTCCTCTATATATATAAAAGTCTGGAGAATACCTTATATCA | P6 Reverse primer to amplify cspB |
| 13 | TTTGTATAAGGATTGGAAAATACCA | P1 Forward Primer to amplify 1000bp upstream SleC |
| 14 | GCAAGATTTAATTTAAAGCTTGATTTAGAACATAAACTCAAAT | P2 Reverse Primer to amplify 1000bp upstream SleC |
| 15 | AATTAAATCTTGCATCAAATCACCCTTTCTTTAAATGAAT | P3 Forward Primer to amplify 1000bp downstream SleC |
| 16 | TGTGTGTCATTTATTATACTTTAAT | P4 Reverse Primer to amplify 1000bp downstream SleC |
| 17 | \| GTATTTATAAATATGAAAAAAGGCGATTTTATATGG \| \| --- \| \|  \| | P5 Forward primer to amplify SleC |
| 18 | CCTTTGGTAATTCTGTCAAATCTATAATCCC | P6 Reverse primer to amplify SleC |
| 19 | AATTTTTTTATCAGGAAACAGCTATGACCG CTAACCATTATTTGATCCATACATA | P1 Forward Primer to amplify 1000bp upstream gerS |
| 20 | TGTATTATGTTCTTGAGAAACTAAGGGGGACTAAAGACAT | P2 Reverse Primer to amplify 1000bp upstream gerS |
| 21 | TCCCCCTTAGTTTCTCAAGAACATAATACATACTATGGTC | P3 Forward Primer to amplify 1000bp downstream gerS |
| 22 | GTAATCATGGTCATATGGATACAGCGGCC TAAATAATAATGTGTATTATCGATA | P4 Reverse Primer to amplify 1000bp downstream gerS |
| 23 | TCTTTTTCTGAGTTATATAGAACTGAAAAGCAGC | P5 Forward primer to amplify gerS |
| 24 | TGCTTAGTTGTGTAGTATAATAAATAATATAATAGATTTGTATATAATATTC | P6 Reverse primer to amplify gerS |
| 25 | AATTTTTTTATCAGGAAACAGCTATGACCG ATACGTTTATAAGTTTCCTTCCATATTAGCGCCTCCT | P1 Forward Primer to amplify 1000bp upstream 32980 |
| 26 | CTACAGCTCCAGTGATTCCTATAGC | P2 Reverse Primer to amplify 1000bp upstream 32980 |
| 27 | TGCTTATTTTCATTGGATAATTGGT | P3 Forward Primer to amplify 1000bp downstream 32980 |
| 28 | GTAATCATGGTCATATGGATACAGCGGCC GCGCTAATATGGAAGGAAACTTATAAACGTATATGTTTAAAATT | P4 Reverse Primer to amplify 1000bp downstream 32980 |
| 29 | CCTTTAGTTCTCCATCTACCCTCATTCTCA | P5 Forward primer to amplify 32980 |
| 30 | CTAAAATTATACCTCCAATTACAGGACCACTCAT | P6 Reverse primer to amplify 32980 |
| 31 | AAATTTTATAAAATAGTTTTATCTACAATTTTTTTATCAGGA | Not diagnostic primer |
| 32 | TAGTggatccTTATTCTACCTCTACCATAAGCATCT | Forward primer to amplify 500 bp upstream 32980 |
| 33 | ATACTcTcgagGGATATTCTGCAATTAATATTTTAAACAGTATAGTTA | Reverse primer to amplify 32980 |
| 34 | ATAggATCCATTTTTGTTTCATGATAACACTCCAATCTT | Forward primer to complement 32980 |
| 35 | CATctcgagTAAACATATACGTTTATAAGTTTTCCTCA | Reverse primer to complement 32980 |
|  |  |  |
